# Metavir 2: new tools for viral metagenome comparison and assembled virome analysis

**DOI:** 10.1186/1471-2105-15-76

**Published:** 2014-03-19

**Authors:** Simon Roux, Jeremy Tournayre, Antoine Mahul, Didier Debroas, François Enault

**Affiliations:** 1 Laboratoire Microorganismes: Génome et Environnement, Clermont Université, Université Blaise Pascal, Clermont-Ferrand, France; 2 CNRS, UMR 6023, LMGE, Aubiere, France; 3 Centre Régional de Ressources Informatiques, Clermont Université, Université Blaise Pascal, Clermont-Ferrand, France

**Keywords:** Virus, Phage, Metagenomics, Web server

## Abstract

**Background:**

Metagenomics, based on culture-independent sequencing, is a well-fitted approach to provide insights into the composition, structure and dynamics of environmental viral communities. Following recent advances in sequencing technologies, new challenges arise for existing bioinformatic tools dedicated to viral metagenome (*i.e*. virome) analysis as (i) the number of viromes is rapidly growing and (ii) large genomic fragments can now be obtained by assembling the huge amount of sequence data generated for each metagenome.

**Results:**

To face these challenges, a new version of Metavir was developed. First, all Metavir tools have been adapted to support comparative analysis of viromes in order to improve the analysis of multiple datasets. In addition to the sequence comparison previously provided, viromes can now be compared through their k-mer frequencies, their taxonomic compositions, recruitment plots and phylogenetic trees containing sequences from different datasets. Second, a new section has been specifically designed to handle assembled viromes made of thousands of large genomic fragments (*i.e*. contigs). This section includes an annotation pipeline for uploaded viral contigs (gene prediction, similarity search against reference viral genomes and protein domains) and an extensive comparison between contigs and reference genomes. Contigs and their annotations can be explored on the website through specifically developed dynamic genomic maps and interactive networks.

**Conclusions:**

The new features of Metavir 2 allow users to explore and analyze viromes composed of raw reads or assembled fragments through a set of adapted tools and a user-friendly interface.

## Background

Viruses are the most abundant biological entities in the biosphere [1] and are now considered as major players in natural ecosystems and their associated cycles and balances [2,3]. Viral communities are known to be mostly composed of new strains [4-6] and are difficult to characterize as (i) most micro-organisms are still impossible to cultivate in the lab for now, hence preventing the culture, isolation and study of their associated viruses and (ii) the absence of a single gene common to all viral genomes prevents the monitoring of uncultured viral diversity using approaches analogous to ribosomal DNA profiling.

Metagenomic approaches, consisting in a random sequencing of the genetic pool isolated from natural samples, circumvent these limitations. Experimental protocols to extract and isolate the encapsidated fraction are now well established [7-9], and viral metagenomes (*i.e*. viromes) have been generated from a broad range of ecosystems. Beyond the description and characterization of the viral genomic diversity, viromes are useful towards more general questions such as biogeography and dispersion of viral particles [10,11], evolution and origin of viruses [12] or epidemiology [13].

Advances in next-generation sequencing and in sequence assembly techniques recently led viral metagenomics a step further, by providing access to large genomic fragments rather than only short reads [14-16]. Indeed, contigs representing complete or near-complete viral genomes were assembled from 454 [17-20] and Illumina HiSeq [21-23] generated viromes. These large assembled sequences (several Kb or tens of Kb, depending on the diversity of the viral community studied) provide access to the genome content and architecture of uncultured viruses and offer the possibility to gain unique insights into the main viral families in the environment.

Two web-servers are currently available for a comprehensive virome analysis: Metavir [24], and Virome [25]. A pipeline (the Viral Metagenome Affiliation Pipeline [26]) was also described but to our knowledge is not available neither as a standalone software or through a web page. Yet, none of these bioinformatic tools were designed for the analysis of assembled datasets and the absence of adapted tools for such assembled viromes was pinpointed as a major bottleneck for viral metagenomic studies [25,27]. Moreover, the growing number of generated viromes calls for the development of comparison strategies to go beyond individual analysis of each dataset. Here, we introduce a new version of Metavir that tackles these two limitations. Metavir 2 includes (i) new ways to compare datasets and (ii) a whole new section which forms the first tool designed for a comprehensive analysis of assembled virome sequences.

## Implementation

### Input and metadata

Registered users can upload their own sequence datasets, either short reads or assembled contigs, in a private space. Input data are checked for being only composed of DNA sequences in fasta format (compressed files in zip, gzip or tar.gz format are accepted). Due to the size of Illumina’s raw datasets (~50 Gb) and computing time required for assembling each dataset, the assembly step cannot be computed through Metavir. Furthermore, a wide range of softwares are available for this step and the choice depends on the type of the sequencing and the nature of the sample: Newbler (454 Life Sciences) is the main software used so far for 454 data [20,28,29], and Illumina data can be assembled with Idba_ud [15], SOAP [30], MetaVelvet [31] or OptiDBA [16].

A set of public viromes is also already available for users to compare with their dataset(s). These viromes are sorted into projects, and linked to the manuscript describing their analysis when available. Various metadata can be added, such as the type of sample from which the virome was sequenced, the location, depth, and temperature of sampling point, and the sequencing technology used to generate the dataset.

### Section 1: tools to analyze raw datasets (unassembled reads)

#### Taxonomic composition

Virome reads are first compared to the complete viral genomes of the RefSeq Virus database using BLAST. The taxonomic composition is then determined using either raw number of best hits or number of best hits normalized by genome length using GAAS [32]. Krona [33] is now used to generate interactive charts representing taxonomic composition of one or more viromes. A custom-designed javascript program has also been implemented to visualize these compositions as interactive heatmaps, with each column representing a dataset and each row a group of viral species. Columns can be switched by mouse drag and drop. Viral species are classified according to the up-to-date NCBI taxonomy, and viral groups can be folded and unfolded with a mouse click.

#### k-mer frequency bias

A virome comparison based on k-mer frequency bias (di-, tri- and tetranucleotides are available) has been implemented as described by Willner and collaborators [34]. Unlike the other available comparison method, based on sequence similarity (generated using reciprocal tBLASTx) and requiring datasets containing at least 50,000 sequences of 100bp, k-mer nucleotide frequencies can be computed for all datasets without size restriction. Briefly, k-mer frequency distribution bias are computed by a custom Perl script and then compared for each pair of viromes. Pairwise euclidian distances between viromes are stored in a matrix, which can be used as input either in a hierarchical clustering or a non-metric multidimensional scaling. Both analysis are computed with R [35] using pvclust [36] and vegan [37] libraries respectively. The non-metric multidimensional scaling (NMDS) is now also available for virome comparison based on sequence similarities, available in Metavir 1.

#### Phylogenetic analyses

To speed up the phylogenetic pipeline, phylogenetic trees are now computed with FastTree [38]. Using the jsPhyloSVG javascript plugin [39], phylogenetic trees are now interactive: they can be displayed as circular or linear, subtrees can be merged, and informations on the origin and affiliation of the sequence of each node can be obtained by clicking on the associated leaf.

#### Individual viral genome recruitment plots

Using the best BLAST hit results against RefseqVirus, each virome sequence with a hit is affiliated to a unique viral genome, *i.e.* each read is recruited by a reference virus. For any selected viral genome, two types of recruitment plots are then available: (i) a scatter plot displaying each recruited read as a dot depending on the position on the genome (on the x-axis) and the identity percentage of the BLAST hit (on the y-axis), and (ii) an histogram presenting the number of recruited reads for each 500-nt long genome part. These plots are generated using the ggplot2 R library [40]. Additional viromes that contain sequences recruited by the selected genomes are also listed and can be added to the current plot. When several datasets are selected, a color is attributed to each virome, used to color dots (in scatter plots) or stacked histograms (in histograms).

### Section 2: assembled viromes annotation and display

#### Contig annotation

Open reading frames (ORFs) are first predicted for each contig through MetaGeneAnnotator [41]. A custom Perl script was designed to detect circular contigs by looking for identical k-mer at the two ends of the sequences. Each circular contig is then trimmed to remove all redundant parts. In order to be able to predict genes spanning the origin of circular contigs, a temporary version of circular contigs is used in the ORF prediction software, in which the first 1,000 nucleotides are duplicated and added at the contig’s end. It has to be noted that this detection of circular contigs will not be effective for contigs computed with assembler like Newbler which already detect and remove such similarity between contig ends.

All predicted translated ORFs are then compared to several databases, namely the RefseqVirus protein database from the NCBI using BLASTp [42], with a threshold of 10^−3^ on e-value, and the PFAM database of protein domains (version 26.0; [43]) using HMMScan [44], with a threshold of 30 on score. A direct comparison of ORFs within a virome is also computed through a BLASTp with the same threshold of 10^−3^ on e-value.

The taxonomic composition and sequence diversity are not calculated the same way for datasets made of long genomic sequences compared to those made of short reads. Using the BLASTp results against reference viruses, three types of taxonomic compositions are computed for each dataset. These compositions are based on (i) best BLAST hit affiliation of each predicted gene, (ii) best BLAST hit affiliation of each contig, and (iii) lowest common ancestor affiliation of each contig. This LCA affiliation is designed to take into account the multiple hits on a single contig: up to five affiliated genes (if available) are considered for each contig, and the affiliation is made at the highest common taxonomy level of the best BLAST hit from these selected genes.

Finally, different clusterings of the predicted ORFs are computed. A global protein sequence clustering with three different thresholds (75, 90 and 98% of similarity) is performed using Uclust [45]. Another clustering is based on protein domain alignments: ORFs are first ordered by size, and used iteratively as a seed for a jackhmmer search [44]. All ORFs recruited by the seed are gathered in a cluster with this seed, and removed from further iterations. Once computed, the domain-based ORFs clusters are affiliated to one or more PFAM domain based on the affiliation of their members. These clusterings are displayed through the rarefaction curve tool, and cluster affiliations can be downloaded in a csv file.

#### Contig display

When an assembled virome is selected, a new “contig maps” page now provides general informations about ORF prediction and contig affiliations, as well as an inset that allows to filter the contig list and access contigs of interest for further analysis (contig maps and networks). This interactive filter, developed using Jquery, let users select contigs based on taxonomic or functional affiliations of predicted genes, and contig size, name or taxonomic affiliation.

An interactive genomic map can be displayed for each contig, this map being drawn using RaphaelSVG and the Raphael-zpd plugin. Each gene affiliation to Refseq viral genomes and PFAM protein domains is indicated when available. Genes can be further investigated as nucleotide and protein sequences are displayed by clicking on the gene either on the map or on the gene table below. Contig annotations can also be downloaded as csv tables, summarized by contig or detailed for each ORFs.

Similarities between contigs and viral genomes and between different contigs can be visualized as an interactive network. In order to take into account all relevant similarities and not only the best BLAST hit for each ORF, all BLAST hits with an e-value lower than 10^−3^ and having a bit-score within a 10% margin from the best BLAST hit bit-score for this ORF are used to build the contig network. In the resulting networks created with Cytoscape-web [46], contigs and reference genomes are represented as nodes, and sequence similarities as edges. Different options are available to customize the network, such as the coloring of edges based on BLAST bit-score, the display of only one edge between two similar contigs or of one edge for each ORFs similarity, or the coloring of genome nodes based on the taxonomy. Another set of filters is also proposed to reduce the number of nodes or edges displayed on screen.

Associated with this network, a contig map comparison tool can be used to display collinearity between contigs and genomes or other contigs selected on the network. This comparisons are displayed through RaphaelSVG and Raphael-zpd. Name and affiliation of each gene is displayed when clicked, and a Jquery pop-up is used to change the sequence order within the plot.

### Common framework

#### Automatic database update

As the RefseqVirus database is quickly growing (40 new genomes are added on average every month), each new release is automatically downloaded and used as the new reference database. Taxonomic composition, gene affiliation (for contig dataset), and recruitment plots of public projects are automatically updated with each release, whereas the update of private projects must be requested by the user.

#### Results and graphics download

All sequence datasets used in a Metavir analysis are available for download in fasta format (affiliated and uncharacterized sequences, sequences included in phylogenetic trees and sequences included in recruitment plots). All tables (taxonomic heatmap, contig and ORF affiliations, results for recruitment analysis) can be downloaded as csv files that can be imported in spreadsheet softwares.

Contig annotations are available in GenBank file format, which can be used in many downstream tools like Artemis [47] or Easyfig [48]. These GenBank files contain the lowest common ancestor affiliation of the contig, as well as the best BLAST hit affiliation of each ORF, the functional annotation of each ORF in PFAM domain, and the sequences of each predicted CDS.

All interactive charts and pictures (contig maps, contig comparisons, phylogenetic trees) can be downloaded in svg format, a publication-ready vectorial format easy to modify using graphics softwares. Static charts generated with R are available to download in pdf and png file format.

Finally, the contig networks can be downloaded in a set of different formats, including graphml and xgmml, ready to be imported in the desktop version of Cytoscape for further analyses and annotations.

### Case study: using metavir to analyze the human gut virome

Two different datasets from the human gut viral community were chosen to illustrate the results that can be obtained with Metavir 2. First, a set of 16 viromes was used to illustrate the section dedicated to unassembled datasets ([49]; project “Human Gut Diet” on Metavir). These metagenomes, sequenced with 454 GS Titanium (884,628 reads of 350 bp/310 Mb), were initially designed to study the dynamics of human gut viral community during a perturbation by a dietary intervention. Two individuals were fed a high fat/low fiber diet (H1 and H2), three were fed a low fat/high fiber (L1, L2 and L3) and one was on an ad-lib diet (X). Samples were collected at up to four time points (days 1, 2, 7 and 8). The second dataset is an assembled virome, resulting from the assembly of Illumina Hi-Seq 2000 reads (5.6 Gb of 100 bp reads) from healthy individuals ([16]; virome “Human gut – All subjects” from project “Human Gut Assembly” on Metavir). This assembled dataset was used here to illustrate the possibilities offered by the new section dedicated to the analysis of contigs.

## Results and discussion

Metavir, a web server dedicated to the analysis of viromes uploaded by registered users, can now be used to analyze the two existing types of datasets: (i) viromes composed of raw reads, mostly generated using pyrosequencing technology and (ii) viromes assembled into contigs, a strategy possible with datasets sequenced with either pyrosequencing or Illumina technology. The novelties of version 2 of Metavir will be illustrated here using both types of datasets (unassembled 454 reads [49] and Illumina assembled contigs [16]), all from human gut samples.

### Additions to the unassembled datasets section

Most published viral metagenomes are still analyzed at the read level. Indeed, pyrosequencing technology is often chosen to generate viromes, as this technology produces long reads and several samples can be easily multiplexed in a single run. Thus, the number of reads in each multiplexed dataset is generally insufficient to produce an assembly. Furthermore, the multiple datasets generated make it possible to study spatial or temporal dynamics in environmental communities [10,22,50-52] or different individuals subjected to different conditions for eukaryote-associated viromes (*e.g.* different diets in [49]). In this context, the comparison of multiple datasets was our major focus while extending the section dedicated to unassembled datasets. In addition to the rarefaction curves and reciprocal tBLASTx comparison available in the initial version of Metavir, taxonomic compositions and phylogenetic analyses can now be used to compare viromes. Furthermore, most of these tools were improved with special attention to the display of results. A brand new tool was also added: the recruitment plot analysis, which makes it possible to accurately study the similarities between virome reads and a viral genome of interest.

#### Taxonomic composition

**Figure 1:**
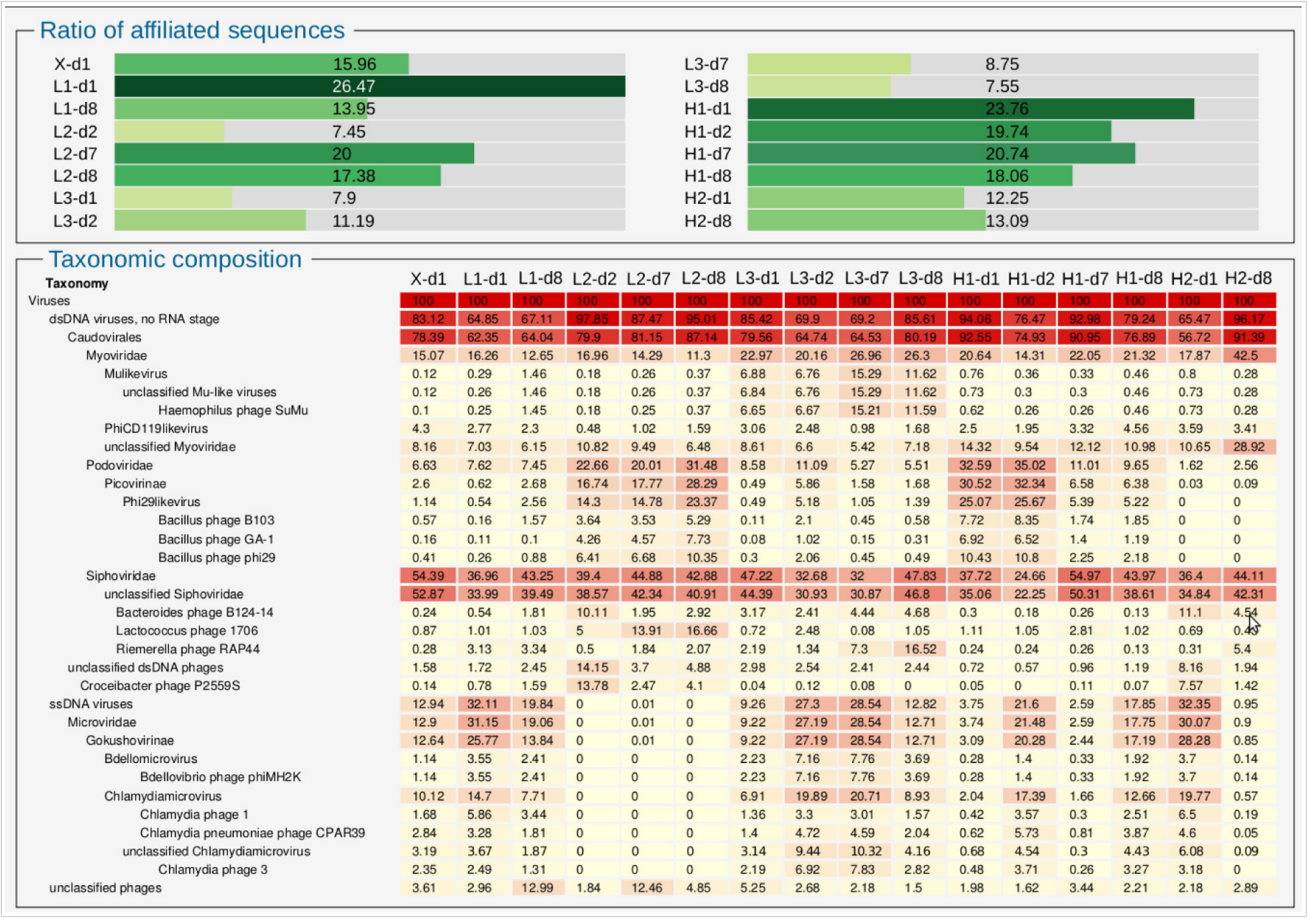
**Taxonomic composition (best hit ratios) of the 16 unassembled datasets from the human gut viromes from Minot *et al*. (**[49]**).** Viral species are classified according to the NCBI taxonomy, and taxonomic groups can be folded or unfolded with a mouse click. Columns have been re-ordered through mouse drag and drop to gather datasets from each subject. Samples are named according to the diet (X: ad-lib diet, H: high fat/low fiber diet, L: low fat/high fiber) of 6 subjects (X, L1, L2, L3, H1, H2) and to the day of the sample collection after the beginning of the experiment (d1, d2, d7 and d8).

Taxonomic composition of viromes is determined by sequence similarity between virome reads and complete known viral genomes, and can be displayed as either raw number of hits or number of hits normalized by genome length [32]. Virome composition can now be visually compared in two ways: (i) merging multiple compositions on the same Krona chart [33] and (ii) an in-house developed interactive heatmap, which allows a more hierarchical view. As an example of the latter, a taxonomic heatmap was generated for the 16 datasets from the human gut (Figure 1). This heatmap allows the user to quickly visualize that these datasets only exhibited similarities with bacteriophages, in accordance with the results presented in Minot *et al.* ([49], Figure two c). Even when the same bacteriophage groups are found in the different datasets, their proportion differ between each virome: *Myoviridae* constitute between 11 and 42% of each virome, *Podoviridae* 2 – 35%, *Siphoviridae* 24 – 55% and *Microviridae* 0 – 31%.

#### k-mer frequency bias

**Figure 2:**
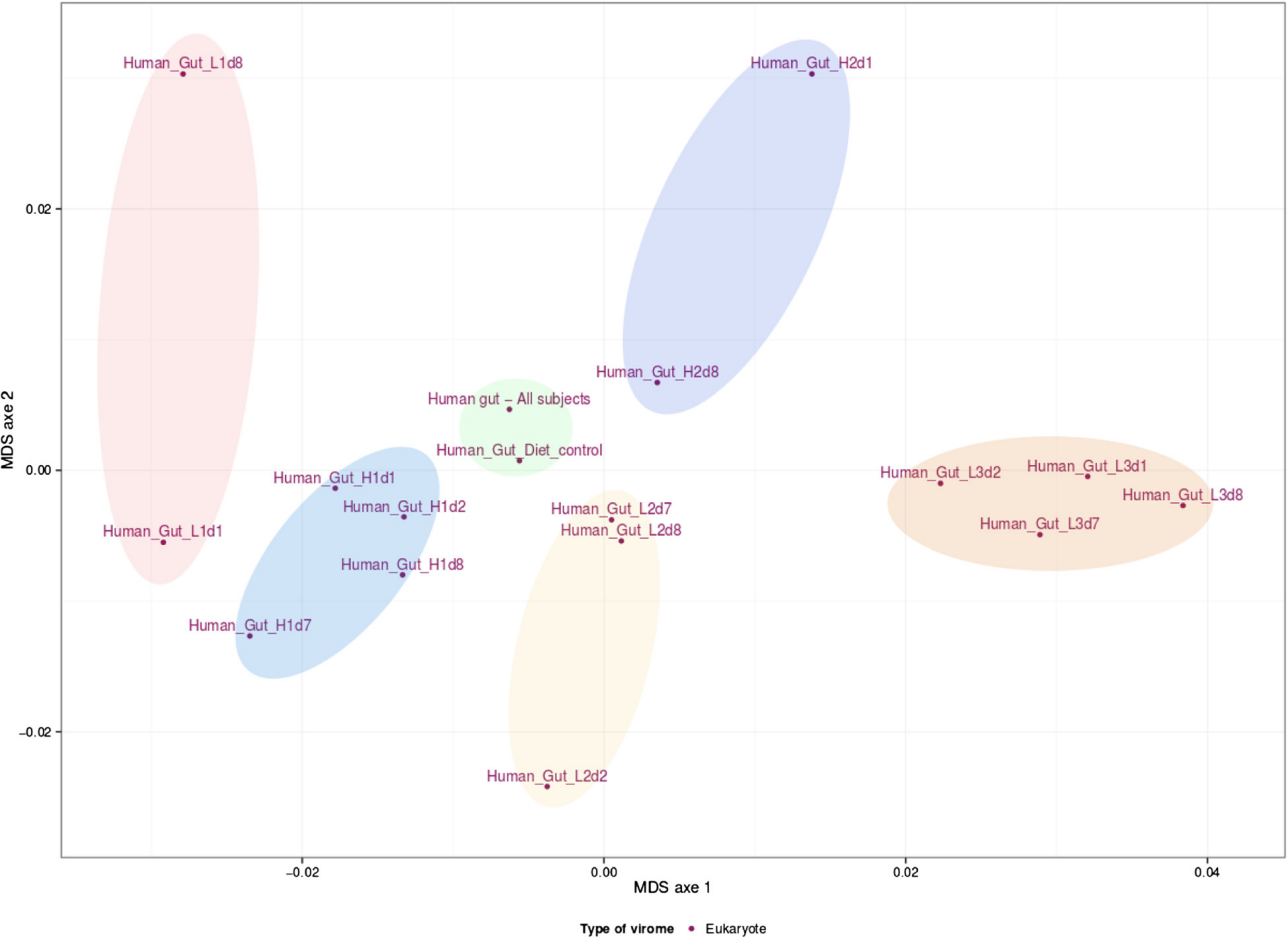
**Comparison of the 16 unassembled human gut viromes and the assembled dataset based on their tetranucleotide compositions.** The NMDS was generated from the pairwise distances computed from the tetranucleotide frequency bias. Each virome is named according to the subject (H1, H2, L1, L2, L3) and day of sampling (day 1, 2, 7 or 8). Samples taken from the same individual are highlighted in shades of blue, yellow and red. Highlighted in green are both the control dataset (X in Figure 1) and the assembled virome described in [16].

A recurrent observation in analyses of virome data is that the majority of reads has no similarity to any known viral sequence [6], as can be noted for human gut viromes (top of Figure 1). Therefore, methods that consider viromes in their entirety rather than only the small fraction affiliated with known sequences are of particular interest. Analysis of k-mer nucleotide frequency bias is such a method and was proved to distinguish viromes from different biomes. This analysis, now available in Metavir, was here applied to the 16 human gut datasets using 4-mer nucleotides (tetranucleotides) and a non-metric multidimensional scaling (Figure 2). Results are again similar to those obtained in Minot *et al*. ([49], Figure five A): even though viral communities seem to be affected by diet (X, H, L), the different samples from each subject (X1, H1, H2, L1, L2 and L3) are gathered indicating that each individual contained a unique virome. However, the k-mer analysis does not support the conclusion that viromes from subjects on the same diet converge over time.

#### Phylogenetic analyses

**Figure 3:**
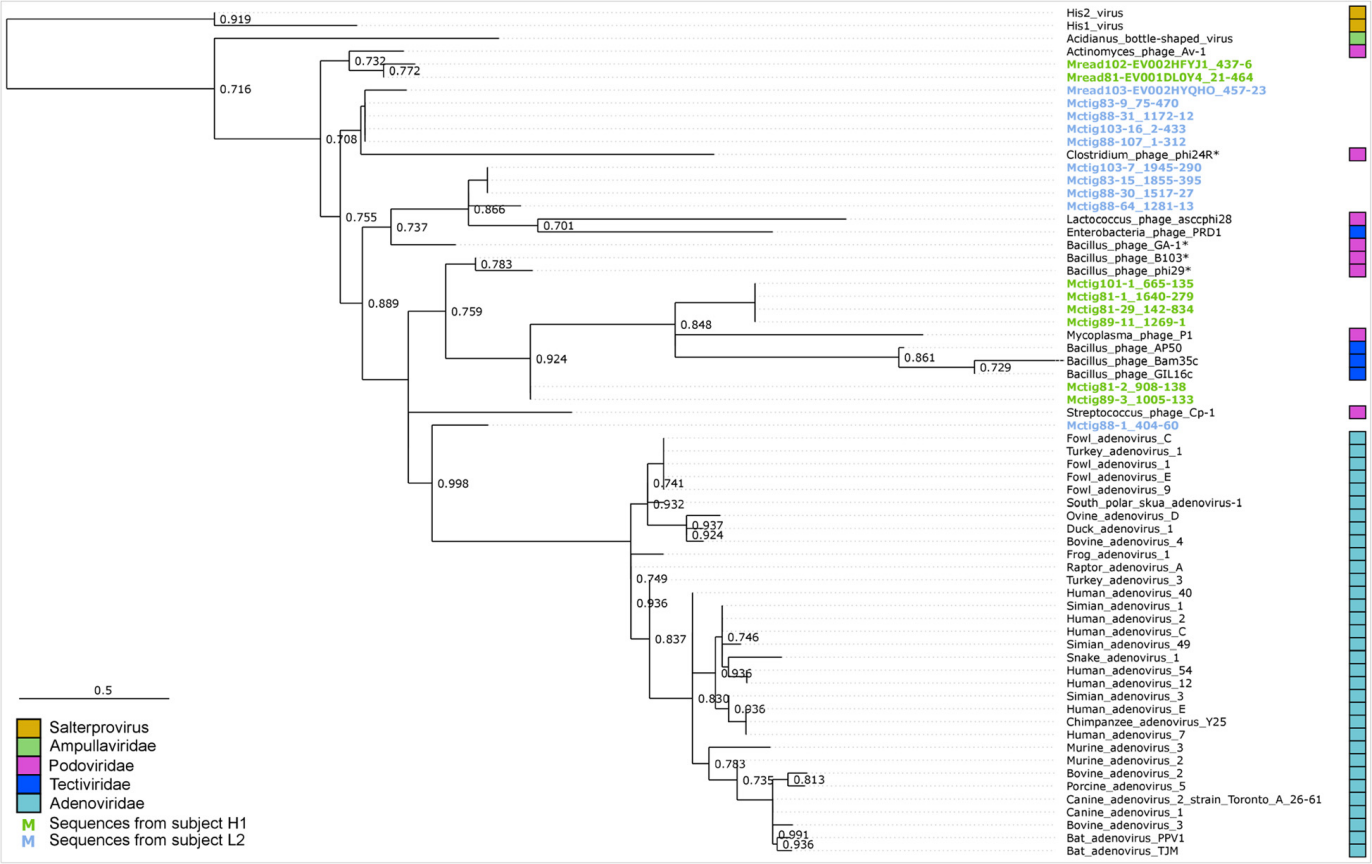
**Phylogenetic tree based on DNA PolB2 sequences (PFAM family PF03175).** All viromes from subjects H1 and L2, for which *Picovirinae* was the most retrieved viral family, were used. Reference sequence names are in black, and sequences from subjects H1 and L2 are highlighted in green and blue respectively. Bootstraps scores greater than 0.70 are indicated on the tree.

Phylogenetic analysis is of particular interest to study specific viral groups and such analysis was implemented in the first version of Metavir [24]. As no gene is common to all viruses, several marker genes are needed to study the major viral groups. The list of markers, initially made of 8 genes, has been expanded to 13 markers, mostly following users’ requests. In Metavir 1, reads from a chosen virome detected as homologous to a selected marker were used to compute a tree including both these virome reads and reference sequences. However, the lack of reference strains close to most environmental viruses limits the efficacy of such analyses and often results in the generation of environmental clades far from references. However, samples from similar biomes often harbor closely related viruses [5,11,52]. To gain a better view of the diversity in each sample and of the relationships between samples, Metavir 2 now offers the opportunity to compute phylogenetic trees that include reads from other viromes. As an example, we conducted such an analysis on the *Picovirinae*, a subfamily of *Podoviridae* (Maximum-likelihood tree computed with FastTree, with default parameters)*.* Indeed, this group is one of the most abundant in 5 of the 16 human gut viromes (Figure 1). A protein primed DNA polymerase, conserved in this family, was used to determine the phylogenetic relationships of the viruses retrieved in these human gut viromes (Figure 3). As expected, all sequences retrieved are most closely related to bacteriophages, and no virome reads appear to be linked to either archeal (*Salterprovirus*) or eukaryotic viruses (*Adenoviridae*). Interestingly, virome sequences from each individual are clustered on the tree, highlighting that the Picovirinae-like phages of subject L2 are distinct from those of H1. Such specificity of viral strains to each individual was noted on a more general scale through virome analysis of genetically linked individuals [28]. In this example, phylogenetic analysis of an abundant viral family confirmed the conclusions drawn from the comparisons of whole viromes.

#### Individual viral genome recruitment plots

**Figure 4:**
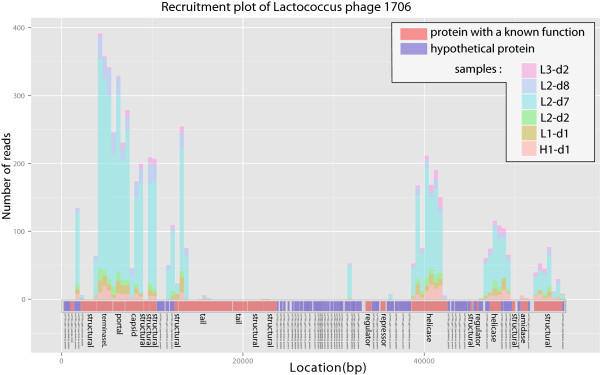
**Recruitment plot of *Lactococcus *phage 1706 for 6 human gut datasets.** Only the 6 viromes with more than 200 reads recruited by this genome were included, *i.e.* reads having their best BLAST hit with this genome. Stacked histograms represent the total number of reads similar to each 500-nt long genome part, with a different color for each virome. Each gene is plotted as a rectangle on the genome map of the Lactococcus phage 1706 at the bottom, with hypothetical proteins in blue, and characterized genes in red. A black frame highlights the three genes involved in host specificity.

Besides the analysis of single reads through BLAST or phylogenetic tools, plots of metagenomic sequences recruited by reference genomes of interest can give a sense of how well this genome is represented in a metagenome (see for example [53]). Indeed, visualizing a chosen genome and the distribution of its associated reads is useful to determine which genes of a known virus are found in an environmental dataset and the similarity level between reference and virome sequences. Recruitment plots can be generated in Metavir, and here again, several datasets can be included in a single plot in order to compare the gene conservation of a virus in different samples. As an example, this tool was here used to further study *Lactococcus* phage 1706, one of the most abundant phages in the 16 datasets from the human gut. As this phage has been isolated from bacteria involved in milk fermentation and not directly from gut microbes, its actual presence in human gut samples is questionable. The plot of virome reads recruited by *Lactococcus* phage 1706 shows that most characterized genes (coding for the main functions of the genome, *i.e*. replication and structure module, highlighted in red on the plot) are retrieved whereas most of the unknown genes (in blue) are not (Figure 4). This suggests that even though phage 1706 is the nearest neighbor of abundant human gut phage(s) in the current state of the reference databases, these gut phages do not have a gene content entirely similar to phage 1706. Furthermore, a gene cassette made of two putative tail proteins and two other structural proteins known to be major players of phage–host specificity in phage 1706 is scarcely retrieved in these datasets ([54]; black frame on Figure four). Thus, it is very likely that the phages retrieved in the human gut viromes, even though similar to this *Lactococcus* phage, infect an alternative host. This example illustrates how recruitment plots help in further understanding the genomic content of environmental viruses and their genomic relatedness with known viruses.

### Analyzing assembled datasets using the new contig section

Even though unassembled viromes proved to be useful for a better characterization of environmental viral communities, long genomic fragments generated through the assembly of metagenomic datasets are usually more informative. Indeed, complete ORFs predicted out of such contig sequences (i) are more often similar to known viruses than short reads [55], (ii) provide more robust phylogenies than using reads representing only a portion of a gene, and (iii) are more appropriate than short random reads in determining the gene content and genetic diversity of a viral community [56]. Moreover, analysis of the genomic content and architecture can provide decisive insights into virus classification and evolution of viral groups [20].

A new section dedicated to the annotation and navigation within sets of contigs has therefore been implemented in Metavir. When assembled viromes, *i.e.* sets of contigs, are uploaded by users, ORFs are predicted [41] and then annotated using sequence similarity results against viral genomes and protein domains. In addition to the general taxonomic composition, contig maps and annotations can be displayed for every contig. As datasets can consist of tens of thousands contigs, users can choose to visualize contigs (i) longer than a defined threshold, (ii) predicted as circular or linear, (iii) affiliated to a particular viral family, and/or (iv) possessing a particular gene. Finally, tools available for read analysis were specifically adapted to assembled datasets: taxonomic compositions are computed using either gene or contig affiliation, phylogenies are generated using predicted ORFs and genetic diversity is computed using either predicted ORFs or domain conservation.

**Figure 5:**
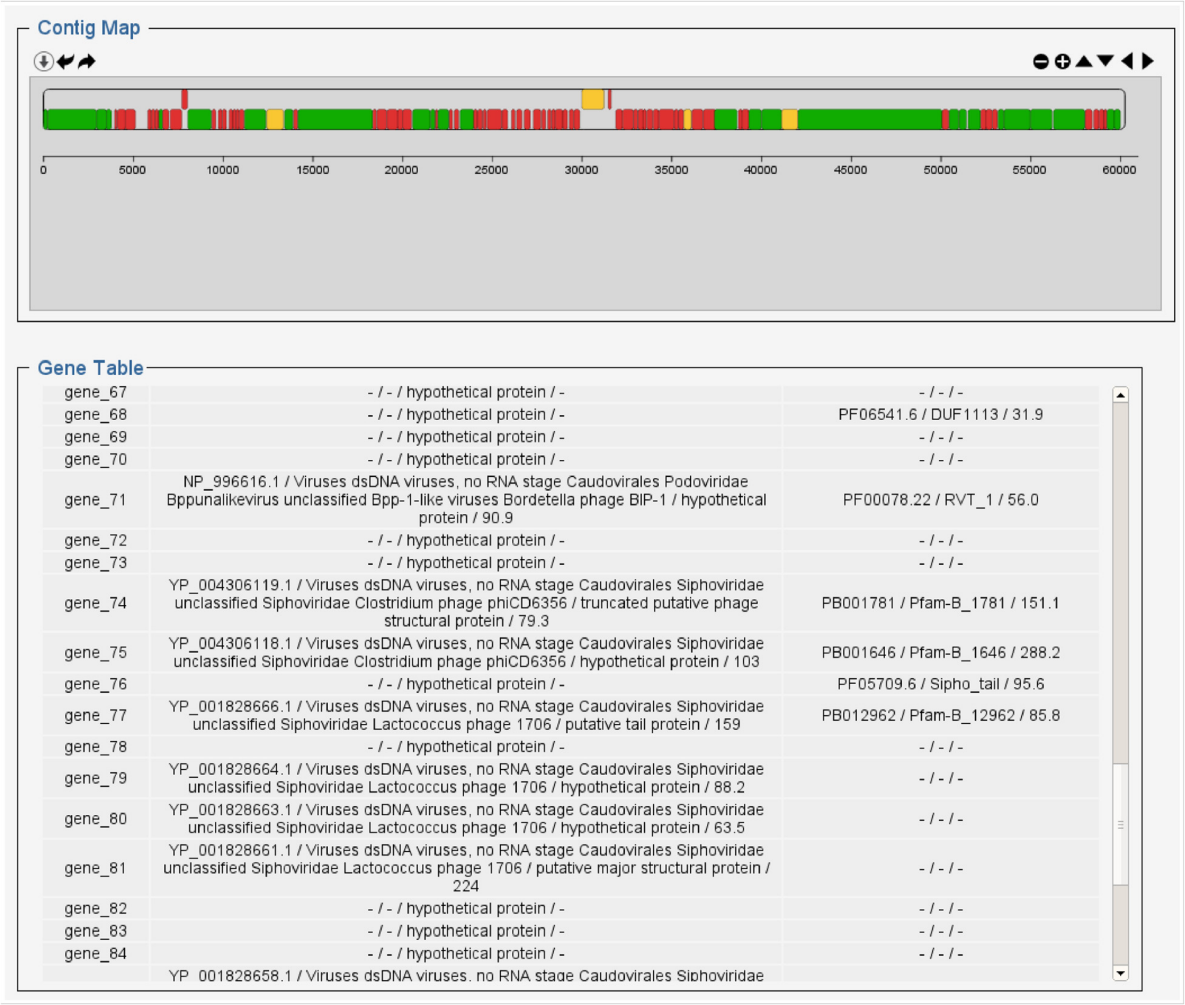
**Automatic annotation of contig 187_43 (complete name: 1470_2012_5M_iter1 _k63_scaffold187_43.0).** On top, a dynamic map displays the predicted genes colored by affiliation (green for genes affiliated to Refseq Virus, yellow when only PFAM affiliation is available, and red for uncharacterized genes). The associated gene table (below) displays for each gene the accession number and annotation of the most similar gene in RefseqVirus and in PFAM (when available).

For the assembled human gut virome used as an example in this section (“Human gut - All subjects” in Metavir), 43,078 ORFs were predicted on the 10,202 uploaded contigs. Furthermore, 60 contigs were predicted as circular and represent potential complete viral genomes. Using the “contig selection” panel, large contigs (>15kb) similar to *Lactococcus* phage 1706 were selected and further examined. For each selected contig, a summary of its annotations is available as an interactive map. The largest sequence (contig_187_43, 60,257 bp) seems to be composed of two sets of genes associated with known viral genomes (green genes at both ends of the contig), whereas a third and central part is made of shorter and uncharacterized genes (red genes) (Figure 5). All genes but three are on the same strand (−), as is generally observed in phage genomes. Moreover, no partial gene is predicted at either end of the sequence, indicating that this contig may represent a complete genome.

**Figure 6:**
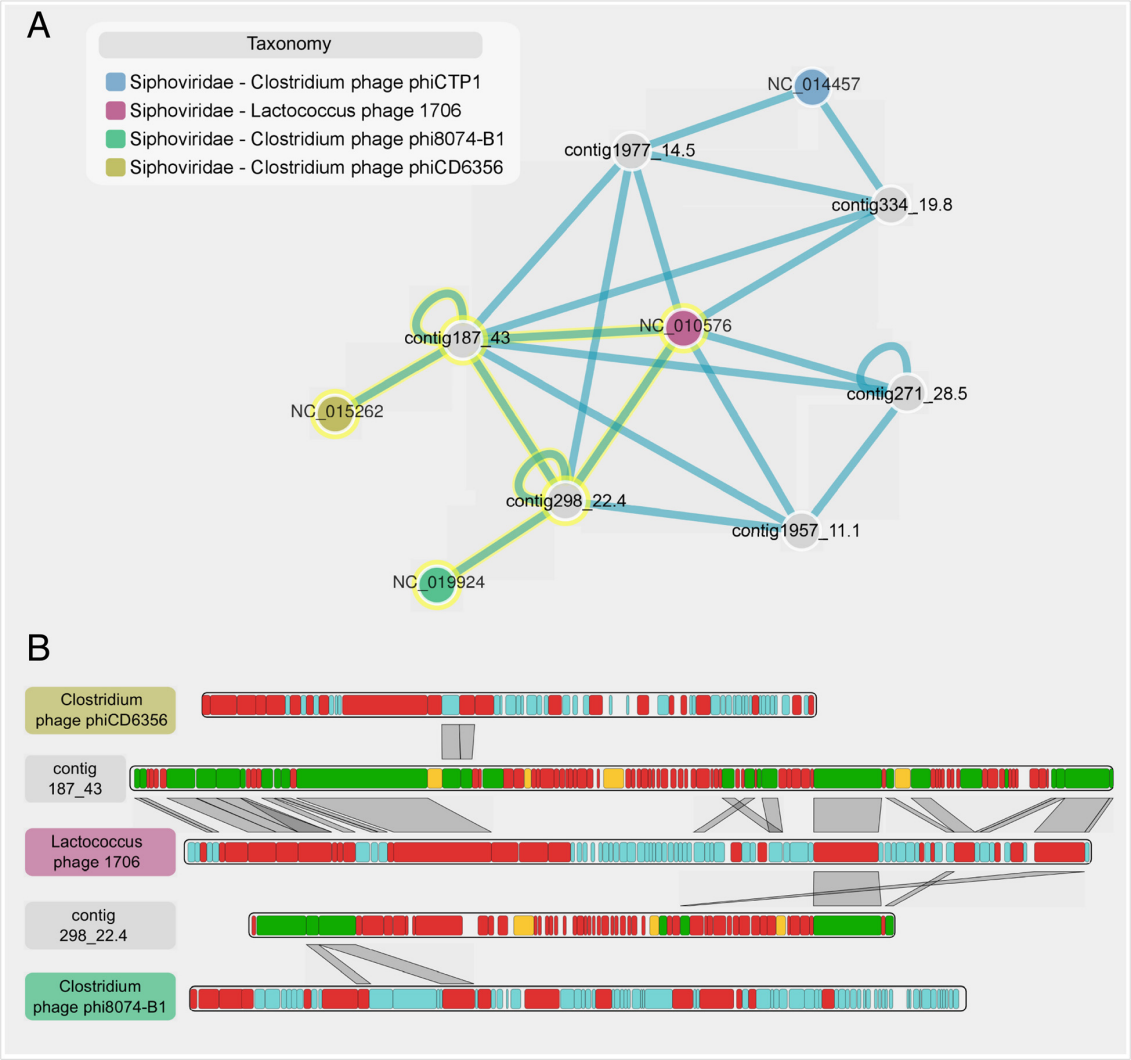
**Contig comparison through network and genome map comparison. A**. Contig network including 6 contigs affiliated to *Lactococcus* phage 1706. Each contig and reference genomes are displayed as nodes, and BLAST similarities are displayed as edges. In this network, we chose to color nodes according to the taxonomy of the reference genomes, and to keep links between nodes only when two genes or more were found to be similar between the two sequences. **B**. Map comparison for contigs and genomes selected in the network (highlighted in yellow in A). The maps of these five selected sequences are vertically stacked, and BLAST hits between genes of two consecutive maps are depicted with gray frames. Sequences were re-ordered to display similarities between *Lactococcus* phage 1706 and the two contigs, as well as similarities between these contigs and *Clostridium* phages. In both network and map comparison, the contig names were simplified: complete name of contig 187_43 is 1470_2012_5M_iter1_k63_scaffold187_43.0, contig 298_22.4 is 1470_2012_5M_iter2_k47_ scaffold298_22.4, contig 334_19.8 is 1470_2012_5M_iter2_k47_scaffold334_19.8, contig 1977_14.5 is 1470_1013_5M_iter6_k39_scaffold1977_14.5, contig 271_28.5 is 1470_2012_5M_iter2_k47_ scaffold271_28.5, and contig 1957_11.1 is 1470_1013_5M_iter6_k39_scaffold1957_11.1.

Relationships between selected contigs and viral references to which they are affiliated can be displayed as an interactive network, where contigs and reference genomes are represented by nodes and sequence similarities as edges. For example, the network containing contigs associated with *Lactococcus* phage 1706 helps to rapidly identify that these contigs are related both to each other and to several *Siphoviridae* genomes (Figure 6A). Contigs and references can then be selected in this network and a genome comparison of the chosen sequences can be displayed. This map-to-map comparison allows the user to identify collinearity between different genomes or genomic fragments. When compared to the complete genome of *Lactococcus* phage 1706, contig_187_43 can definitely be considered as a putative complete genome closely related to this phage, as both their sizes and gene organizations are very similar (Figure 6B). Interestingly, the similarities between this contig and *Clostridium* phage phiCD6356 are limited to two genes which are part of the host-associated cassette previously discussed. Thus, contig_187_43 likely originates from a phage closely related to *Lactococcus* phage 1706, but which could instead infect members of the *Clostridium* genus. The second contig displayed on Figure 6B, contig_289_22.4, only shares one core gene module with phage 1706 and harbors several similarities to a distinct *Clostridium* phage. These two contigs, that both exhibit similarities to *Lactococcus* phage 1706, are here shown to be heterogeneous in nature. Furthermore, genes of contig_187_43 similar to *Lactococcus* phage 1706 correspond to the genes frequently retrieved in unassembled datasets (Figure 4), indicating that this contig might represent a prevalent virotype of the human gut. This genomic analysis of large assembled sequences exemplifies how such datasets can provide further insights into viral communities and viral species.

## Conclusion

This new release of Metavir provides a wide range of tools to analyze either raw or assembled viral metagenomes in a comprehensive way. As virome projects now regularly encompass multiple samples and as more and more viromes are being published, a special effort was made towards virome comparison. Two new large scale methods were implemented and all existing Metavir tools were modified so that they can be used to compare datasets. Furthermore, a new section has been specifically developed to handle sets of large genomic contigs. As these datasets can be large and as all individual sequences can be of interest, we paid special attention to the interface, with filtering panels and network visualization. Selected contigs can then be analyzed in detail by comparing their automatic annotations in terms of gene content and genomic maps. Finally, with its extended or new tools and sections, Metavir 2 provides a comprehensive framework with a user-friendly interface to explore any kind of viromes, and should help virologists to make the most of their metagenomics data.

## Availability and requirements

**Project Name**: Metavir

**Project home page**: http://metavir-meb.univ-bpclermont.fr

**Operating system(s)**: Platform independent

**Programming language**: Perl, Php, Javascript, Css, R

**Other requirements:** Javascript installed on user side

**Licence**: GNU GPL3

**Any restrictions to use by non-academics**: No.

## Competing interests

The authors declare that they have no competing interests.

## Authors’ contributions

SR, DD and FE designed the tools. SR, JT and AM developed the different scripts. SR and FE wrote the manuscript. All authors read and approved the final manuscript.

## References

[B1] Suttle CA Viruses in the sea Nature 2005 437 356 361 10.1038/nature04160 16163346

[B2] Suttle CA Marine viruses–major players in the global ecosystem Nat Rev Microbiol 2007 5 801 812 10.1038/nrmicro1750 17853907

[B3] Rohwer F Thurber RV Viruses manipulate the marine environment Nature 2009 459 207 212 10.1038/nature08060 19444207

[B4] Hatfull GF Hendrix RW Bacteriophages and their Genomes Curr Opin Virol 2011 1 298 303 10.1016/j.coviro.2011.06.009 22034588 PMC3199584

[B5] Roux S Enault F Robin A Ravet V Personnic S Theil S Colombet J Sime-Ngando T Debroas D Assessing the diversity and specificity of two freshwater viral communities through metagenomics PLoS One 2012 7 e33641 10.1371/journal.pone.0033641 22432038 PMC3303852

[B6] Edwards RA Rohwer F Viral metagenomics Nat Rev Microbiol 2005 3 504 510 10.1038/nrmicro1163 15886693

[B7] Duhaime MB Sullivan MB Ocean viruses: rigorously evaluating the metagenomic sample-to-sequence pipeline Virology 2012 434 181 186 10.1016/j.virol.2012.09.036 23084423

[B8] Vega Thurber R Haynes M Breitbart M Wegley L Rohwer F Laboratory procedures to generate viral metagenomes Nat Protoc 2009 4 470 483 10.1038/nprot.2009.10 19300441

[B9] Willner D Hugenholtz P From deep sequencing to viral tagging: Recent advances in viral metagenomics BioEssays 2013 35 436 442 10.1002/bies.201200174 23450659

[B10] Fancello L Trape S Robert C Boyer M Popgeorgiev N Raoult D Desnues C Viruses in the desert: a metagenomic survey of viral communities in four perennial ponds of the Mauritanian Sahara ISME J 2013 7 359 369 10.1038/ismej.2012.101 23038177 PMC3554411

[B11] Whon TW Kim M-S Roh SW Shin N-R Lee H-W Bae J-W Metagenomic characterization of airborne viral DNA diversity in the near-surface atmosphere J Virol 2012 86 8221 8331 10.1128/JVI.00293-12 22623790 PMC3421691

[B12] Kristensen DM Mushegian AR Dolja VV Koonin EV New dimensions of the virus world discovered through metagenomics Trends Microbiol 2010 18 11 19 10.1016/j.tim.2009.11.003 19942437 PMC3293453

[B13] Palacios G Druce J Du L Tran T Birch C Briese T Conlan S Quan P Hui J Marshall J Simons JF Egholm M Paddock CD Shieh W Goldsmith CS Zaki SR Catton M Lipkin WI A new arenavirus in a cluster of fatal transplant-associated diseases N Engl J Med 2008 358 991 998 10.1056/NEJMoa073785 18256387

[B14] Koren S Treangen TJ Pop M Bambus 2: scaffolding metagenomes Bioinformatics 2011 27 2964 2971 10.1093/bioinformatics/btr520 21926123 PMC3198580

[B15] Peng Y Leung HCM Yiu SM Chin FYL IDBA-UD: a de novo assembler for single-cell and metagenomic sequencing data with highly uneven depth Bioinformatics 2012 28 1420 1428 10.1093/bioinformatics/bts174 22495754

[B16] Minot S Wu GD Lewis JD Bushman FD Conservation of gene cassettes among diverse viruses of the human Gut PLoS One 2012 7 e42342 10.1371/journal.pone.0042342 22900013 PMC3416800

[B17] Ng TFF Willner DL Lim YW Schmieder R Chau B Nilsson C Anthony S Ruan Y Rohwer F Breitbart M Broad surveys of DNA viral diversity obtained through viral metagenomics of mosquitoes PLoS One 2011 6 e20579 10.1371/journal.pone.0020579 21674005 PMC3108952

[B18] Rosario K Duffy S Breitbart M Diverse circovirus-like genome architectures revealed by environmental metagenomics J Gen Virol 2009 90 2418 2424 10.1099/vir.0.012955-0 19570956

[B19] Diemer GS Stedman KM A novel virus genome discovered in an extreme environment suggests recombination between unrelated groups of RNA and DNA viruses Biol Direct 2012 7 13 10.1186/1745-6150-7-13 22515485 PMC3372434

[B20] Roux S Krupovic M Poulet A Debroas D Enault F Evolution and diversity of the Microviridae viral family through a collection of 81 new complete genomes assembled from virome reads PLoS One 2012 7 e40418 10.1371/journal.pone.0040418 22808158 PMC3394797

[B21] Coetzee B Freeborough M-J Maree HJ Celton J-M Rees DJG Burger JT Deep sequencing analysis of viruses infecting grapevines: virome of a vineyard Virology 2010 400 157 163 10.1016/j.virol.2010.01.023 20172578

[B22] Emerson JB Thomas BC Andrade K Allen EE Heidelberg KB Banfield JF Metagenomic assembly reveals dynamic viral populations in hypersaline systems Appl Environ Microbiol 2012 78 6309 6320 10.1128/AEM.01212-12 22773627 PMC3416638

[B23] Minot S Grunberg S Wu GD Lewis JD Bushman FD Hypervariable loci in the human gut virome Proc Natl Acad Sci USA 2012 109 3962 3966 10.1073/pnas.1119061109 22355105 PMC3309749

[B24] Roux S Faubladier M Mahul A Paulhe N Bernard A Debroas D Enault F Metavir: a web server dedicated to virome analysis Bioinformatics 2011 27 3074 3075 10.1093/bioinformatics/btr519 21911332

[B25] Wommack KE Bhavsar J Polson SW Chen J Dumas M Srinivasiah S Furman M Jamindar S Nasko DJ VIROME: a standard operating procedure for analysis of viral metagenome sequences Stand Genomic Sci 2012 6 427 439 10.4056/sigs.2945050 23407591 PMC3558967

[B26] Lorenzi HA Hoover J Inman J Safford T Murphy S Kagan L Williamson SJ TheViral MetaGenome Annotation Pipeline (VMGAP): an automated tool for the functional annotation of viral Metagenomic shotgun sequencing data Stand Genomic Sci 2011 4 418 429 10.4056/sigs.1694706 21886867 PMC3156399

[B27] Fancello L Raoult D Desnues C Computational tools for viral metagenomics and their application in clinical research Virology 2012 434 162 174 10.1016/j.virol.2012.09.025 23062738 PMC7111993

[B28] Reyes A Haynes M Hanson N Angly FE Heath AC Rohwer F Gordon JI Viruses in the faecal microbiota of monozygotic twins and their mothers Nature 2010 466 334 338 10.1038/nature09199 20631792 PMC2919852

[B29] Ray J Dondrup M Modha S Steen IH Sandaa R-A Clokie M Finding a needle in the virus metagenome haystack–micro-metagenome analysis captures a snapshot of the diversity of a bacteriophage armoire PLoS One 2012 7 e34238 10.1371/journal.pone.0034238 22509283 PMC3324506

[B30] Li R Li Y Kristiansen K Wang J SOAP: short oligonucleotide alignment program Bioinformatics 2008 24 713 714 10.1093/bioinformatics/btn025 18227114

[B31] Namiki T Hachiya T Tanaka H Sakakibara Y MetaVelvet: an extension of Velvet assembler to de novo metagenome assembly from short sequence reads Nucleic Acids Res 2012 40 e155 10.1093/nar/gks678 22821567 PMC3488206

[B32] Angly FE Willner D Prieto-Davó A Edwards RA Schmieder R Vega-Thurber R Antonopoulos DA Barott K Cottrell MT Desnues C Dinsdale EA Furlan M Haynes M Henn MR Hu Y Kirchman DL McDole T McPherson JD Meyer F Miller RM Mundt E Naviaux RK Rodriguez-Mueller B Stevens R Wegley L Zhang L Zhu B Rohwer F The GAAS metagenomic tool and its estimations of viral and microbial average genome size in four major biomes PLoS Comput Biol 2009 5 e1000593 10.1371/journal.pcbi.1000593 20011103 PMC2781106

[B33] Ondov BD Bergman NH Phillippy AM Interactive metagenomic visualization in a Web browser BMC Bioinformatics 2011 12 385 10.1186/1471-2105-12-385 21961884 PMC3190407

[B34] Willner D Thurber RV Rohwer F Metagenomic signatures of 86 microbial and viral metagenomes Environ Microbiol 2009 11 1752 1756 10.1111/j.1462-2920.2009.01901.x 19302541

[B35] R Core Team R: A Language and Environment for Statistical Computing 2013 Vienna, Austria: R Foundation for Statistical Computing

[B36] Suzuki R Shimodaira H Pvclust: an R package for assessing the uncertainty in hierarchical clustering Bioinformatics 2006 22 1540 1542 10.1093/bioinformatics/btl117 16595560

[B37] Oksanen J Kindt R Legendre P O’Hara B Simpson GL Solymos P Stevens MHH Wagner H The vegan Package 2008

[B38] Price MN Dehal PS Arkin AP FastTree 2–approximately maximum-likelihood trees for large alignments PLoS One 2010 5 e9490 10.1371/journal.pone.0009490 20224823 PMC2835736

[B39] Smits SA Ouverney CC jsPhyloSVG: a javascript library for visualizing interactive and vector-based phylogenetic trees on the web PLoS One 2010 5 e12267 10.1371/journal.pone.0012267 20805892 PMC2923619

[B40] Wickham Hggplot2: Elegant Graphics for Data Analysis 2009 New York, NY 10036: Springer Publishing Company

[B41] Noguchi H Taniguchi T Itoh T MetaGeneAnnotator: detecting species-specific patterns of ribosomal binding site for precise gene prediction in anonymous prokaryotic and phage genomes DNA Res 2008 15 387 396 10.1093/dnares/dsn027 18940874 PMC2608843

[B42] Altschul SF Gish W Miller W Myers EW Lipman DJ Basic local alignment search tool J Mol Biol 1990 215 403 410 10.1016/S0022-2836(05)80360-2 2231712

[B43] Punta M Coggill PC Eberhardt RY Mistry J Tate J Boursnell C Pang N Forslund K Ceric G Clements J Heger A Holm L Sonnhammer ELL Eddy SR Bateman A Finn RD The Pfam protein families database Nucleic Acids Res 2012 40 D290 D301 10.1093/nar/gkr1065 22127870 PMC3245129

[B44] Eddy SR Accelerated profile HMM searches PLoS Comput Biol 2011 7 e1002195 10.1371/journal.pcbi.1002195 22039361 PMC3197634

[B45] Edgar RC Search and clustering orders of magnitude faster than BLAST Bioinformatics 2010 26 2460 2461 10.1093/bioinformatics/btq461 20709691

[B46] Lopes CT Franz M Kazi F Donaldson SL Morris Q Bader GD Cytoscape Web: an interactive web-based network browser Bioinformatics 2010 26 2347 2348 10.1093/bioinformatics/btq430 20656902 PMC2935447

[B47] Rutherford K Parkhill J Crook J Horsnell T Barrell B Rice P Artemis: sequence visualization and annotation Bioinformatics 2000 16 944 945 10.1093/bioinformatics/16.10.944 11120685

[B48] Sullivan MJ Petty NK Beatson SA Easyfig: a genome comparison visualizer Bioinformatics 2011 27 1009 1010 10.1093/bioinformatics/btr039 21278367 PMC3065679

[B49] Minot S Sinha R Chen J Li H Keilbaugh SA Wu GD Lewis JD Bushman FD The human gut virome: inter-individual variation and dynamic response to diet Genome Res 2011 21 1616 1625 10.1101/gr.122705.111 21880779 PMC3202279

[B50] Angly FE Felts B Breitbart M Salamon P Edwards RA Carlson C Chan AM Haynes M Kelley S Liu H Mahaffy JM Mueller JE Nulton J Olson R Parsons R Rayhawk S Suttle CA Rohwer F The marine viromes of four oceanic regions PLoS biology 2006 4 e368 10.1371/journal.pbio.0040368 17090214 PMC1634881

[B51] Rosario K Nilsson C Lim YW Ruan Y Breitbart M Metagenomic analysis of viruses in reclaimed water Environ Microbiol 2009 11 2806 2820 10.1111/j.1462-2920.2009.01964.x 19555373

[B52] Yoshida M Takaki Y Eitoku M Nunoura T Takai K Metagenomic analysis of viral communities in (hado) pelagic sediments PLoS One 2013 8 e57271 10.1371/journal.pone.0057271 23468952 PMC3584133

[B53] Ghai R Martin-Cuadrado A-B Molto AG Heredia IG Cabrera R Martin J Verdú M Deschamps P Moreira D López-García P Mira A Rodriguez-Valera F Metagenome of the Mediterranean deep chlorophyll maximum studied by direct and fosmid library 454 pyrosequencing ISME J 2010 4 1154 1166 10.1038/ismej.2010.44 20393571

[B54] Garneau JE Tremblay DM Moineau S Characterization of 1706, a virulent phage from *Lactococcus lactis* with similarities to prophages from other Firmicutes Virology 2008 373 298 309 10.1016/j.virol.2007.12.002 18191977

[B55] Wommack KE Bhavsar J Ravel J Metagenomics: read length matters Appl Environ Microbiol 2008 74 1453 1463 10.1128/AEM.02181-07 18192407 PMC2258652

[B56] Hurwitz BL Sullivan MB The Pacific Ocean Virome (POV): a marine viral metagenomic dataset and associated protein clusters for quantitative viral ecology PLoS One 2013 8 e57355 10.1371/journal.pone.0057355 23468974 PMC3585363

